# Optimization of Internet-Delivered Cognitive Behavioral Therapy for Canadian Leaders Within Public Safety: Qualitative Study

**DOI:** 10.2196/72321

**Published:** 2025-04-17

**Authors:** Jill AB Price, Hugh C McCall, Sam A Demyen, Shaylee M Spencer, Benjamin MW Katz, Alyssa P Clairmont, Heather D Hadjistavropoulos

**Affiliations:** 1 Canadian Institute for Public Safety Research and Treatment Regina, SK Canada; 2 PSPNET University of Regina Regina, SK Canada; 3 Department of Psychology University of Regina Regina, SK Canada

**Keywords:** first responders, public safety personnel, leadership, mental health, digital mental health interventions, internet-delivered cognitive behavioral therapy

## Abstract

**Background:**

Canadian public safety personnel (PSP) report high rates of mental health concerns and barriers to treatment. PSPNET is a clinical research unit that offers internet-delivered cognitive behavioral therapy (ICBT) that is free, confidential, and developed with and for PSP. Treatment outcomes are promising with clinically significant symptom improvement (eg, anxiety, depression, and posttraumatic stress) and favorable treatment satisfaction. While these results are promising, research has yet to explore ways to optimize therapist-guided ICBT for leaders within public safety. Optimizing ICBT for leaders is particularly important given their widespread organizational impact.

**Objective:**

This study aims to investigate (1) the perceived mental health stressors of Canadian leaders within public safety, (2) the degree to which leaders perceived existing therapist-guided ICBT courses tailored for PSP (ie, *PSP Wellbeing Course* and *PSP PTSD Course*) as suitable for their needs, and (3) ways to further optimize therapist-guided ICBT for public safety leaders.

**Methods:**

This study included 10 clients who self-identified as being in a supervisory or leadership position within their public safety organization and completed either the therapist-guided *PSP Wellbeing Course* or *PSP PTSD Course*. We used descriptive statistics to analyze demographics, mental health symptoms, treatment engagement, and treatment satisfaction. We also used a reflexive thematic analysis of semistructured interview transcripts to assess leaders’ course perceptions and feedback.

**Results:**

Canadian leaders within public safety reported occupational and nonoccupational stressors and enrolled in ICBT to support their own or colleagues’ mental health. Most clients enrolled in the *PSP Wellbeing Course*, accessed 4 of 5 lessons (n=7, 70%), engaged with therapist support (n=7, 70%), and identified as employed (n=8, 80%), White (n=8, 80%), and men (n=7, 70%) with an average age of 45 years. At pretreatment, 80% of clients endorsed clinically significant symptoms of one or more disorders; most often depression (n=7, 70%) and anger (n=6, 60%). Clients reported favorable attitudes toward the ICBT courses with most reporting that they were satisfied with the course (n=9, 90%). Feedback to further optimize ICBT content for leaders included the development of a leader case story (n=6, 60%) and new resources to help leaders apply skills learned in ICBT within the context of their leadership roles (n=4, 40%). Leaders also recommended optimizing ICBT delivery by improving the platform technology and incorporating more multimedia.

**Conclusions:**

Canadian leaders within public safety perceived therapist-guided ICBT developed with and for PSP as a suitable treatment option for their needs and identified ways to further optimize its content and delivery. Future research should investigate the impacts of these efforts and explore optimizing ICBT for other groups of clients.

**Trial Registration:**

ClinicalTrials.gov NCT04127032, https://www.clinicaltrials.gov/study/NCT04127032; ClinicalTrials.gov NCT04335487, https://clinicaltrials.gov/study/NCT04335487

## Introduction

### Background

Public safety personnel (PSP) are individuals whose work protects, supports, and secures the safety of citizens within border services, communications (eg, dispatch), corrections, emergency managers, fire (career and volunteer), intelligence, paramedics, policing (self-administered, municipal, provincial, and federal), and search and rescue, among others [1]. PSP report high rates of mental health challenges; in a survey of 5813 Canadian PSP, 44.5% reported clinically significant symptoms of one or more mental disorders [2]. PSP also experience barriers to accessing mental health care, including structural barriers (eg, financial constraints, limited availability of services, distance from services, inconvenience of seeking treatment) and attitudinal barriers (eg, concerns about stigma, the low perceived need for help) [3,4].

### Occupational Stressors

Occupational stressors refer to workplace challenges that impact mental health [1]. PSP’s occupational stressors have been categorized into operational stressors, which are directly tied to tasks in the workplace (eg, potentially psychologically traumatic events [PPTEs], job-related injuries, infringement on other parts of life, and excessive demands) and organizational stressors, which are associated with the setting or context of the job (eg, staff shortages, lack of training and resources, and inconsistent leadership) [1,5-8]. PPTEs refer to events that have the potential to induce symptoms of posttraumatic stress disorder (PTSD) or related disorders (eg, events involving death, serious injury, sexual violence) [1]. Among Canadian PSP, PPTEs are both common and predictive of symptoms of various mental health challenges [5].

### Leaders’ Mental Health

The term “leader” refers to an individual in a supervisory or management position in their workplace [9]. Despite perceptions that leaders are more resilient than nonleaders [10], have greater access to mental health supports [10,11], have greater work-life balance, and have more control over their work schedule and demands [12], mental health problems appear to be highly prevalent among leaders in the general public [13,14]. Campbell et al [13] found that 88% of leaders reported that their job is the main source of stress in their lives, 79% reported increased stress since taking on a supervisory or management position, and over 60% claimed that their organization was not providing sufficient mental health support. Research similarly shows that workplace leaders experience greater occupational stressors than staff in nonleadership roles [15]. Compared with nonleaders, leaders are also more likely to get inadequate amounts of sleep (less than 6 hours) and self-report consuming more alcohol at work, both of which can predict disruptive and negative leadership behaviors and worsen preexisting symptoms of mental disorders [10].

Despite the high rates of mental health concerns among PSP and the unique stressors faced by organizational leaders, relatively little is known about the unique challenges faced by PSP in leadership roles. This is an important gap in the research literature because mental health concerns among PSP leaders are not only inherently distressing but also have important implications for the mental health and well-being of PSP in nonleadership roles. Research shows that mental health challenges among leaders—within the general public or public safety—can have widespread organizational impacts that affect nonleaders’ job performance and satisfaction [10,14,16-18]. Mental health challenges can negatively impact leaders’ behavioral functioning (eg, aggression and sleep) and cognitive functioning (eg, concentration and decision-making), and there is evidence that emotional difficulties among leaders in the general population (eg, anger, anxiety, and depression) predict abusive management practices and unreasonable expectations of staff [10]. PSP further report that their experiences of distress and burnout are impacted by the quality of relationships with their leaders [17]. It is important to note that leaders’ mental health is also affected by the mental health and morale of their staff [10], highlighting a need for mental health support among PSP in leadership and nonleadership roles alike.

### Internet-Delivered Cognitive Behavioral Therapy

Internet-delivered cognitive behavioral therapy (ICBT) is an evidence-based treatment for various mental health symptoms (eg, anger, anxiety, depression, and posttraumatic stress) that has demonstrated excellent outcomes among the general public [19,20] and Canadian PSP [21,22]. ICBT typically consists of self-directed learning of evidence-based treatment principles in web-based modules, often accompanied by therapist support via email or phone. This web-based, largely self-directed format affords privacy and convenience, can allow therapists to treat more clients at a given time, is cost-effective, and can, in turn, help overcome several barriers to treatment (eg, distance from services, cost of treatment, and privacy concerns) [23,24].

PSPNET is a clinical research unit located at the University of Regina in Saskatchewan, Canada, which was founded in response to the Government of Canada’s National Action Plan on Posttraumatic Stress Injuries [25]. PSPNET provides free, confidential, and evidence-based ICBT developed with and for Canadian PSP. PSPNET offers a range of ICBT courses, including courses with or without optional therapist support, PTSD-specific and transdiagnostic courses (ie, courses designed to treat symptoms of various mental disorders), courses in English and French, and courses for both PSP and their spouses or significant others. PSPNET’s research has shown that Canadian PSP, in general, report favorable perceptions of ICBT [3,18,26,27] as well as good treatment engagement, symptom reduction, and treatment satisfaction [22,28-31]. Although there is some evidence that leaders within public safety have favorable perceptions of ICBT [18,32], little is known about their unique mental health stressors and how ICBT might be further optimized to address those stressors. Interviews were invaluable in PSPNET’s early efforts to tailor ICBT for PSP, but to date, interviews have not been conducted specifically with leaders to gain insight into their unique challenges, experiences, and treatment preferences [26].

### Objectives and Hypotheses

This study explored ways to optimize ICBT for Canadian leaders within public safety by investigating: (1) leaders’ mental health stressors, (2) the degree to which leaders perceived existing therapist-guided ICBT programs (ie, *PSP Wellbeing Course* and *PSP PTSD Course*, both described in later sections) tailored for PSP as suitable for their unique needs, and (3) leaders’ feedback on how to further optimize therapist-guided ICBT to better meet their treatment needs. We made 3 hypotheses. First, consistent with research showing high rates of various mental health problems among PSPNET clients [21,22] and Canadian PSP more broadly [2], we hypothesized that PSP leaders would report a variety of mental health concerns. Second, consistent with research showing positive perceptions of ICBT among PSPNET clients [21,22,28,30], Canadian PSP leaders [18,32], and Canadian PSP at varying levels of leadership [3,18,26], we hypothesized that participants in the current study would report largely positive perceptions of therapist-guided ICBT. Third, consistent with our past research using qualitative analysis of interviews with PSP to identify areas for improvement in ICBT [26,28,33,34], we hypothesized that participants would provide tangible feedback to further optimize therapist-guided ICBT.

## Methods

### Ethical Considerations

This qualitative study was approved by the Research Ethics Board at the University of Regina (#2019-157) and was carried out within the context of a longitudinal single-group open observational trial (NCT04127032) and open cohort preference trial (NCT04335487) registered on Clinicaltrials.gov. Clients provided informed consent after being made aware of the study details and the potential benefits and risks of participation. Clients were provided access to the *PSP Wellbeing Course* or *PSP PTSD Course* for up to 1 year but were not offered incentives to encourage participation. Client data were stored on a secure server and were deidentified prior to analyses.

### Study Design

Prospective clients signed up for a PSPNET account, provided informed consent to participate, and completed eligibility screening questionnaires that collected demographic, clinical, and occupational information. This web-based screening was followed by a telephone interview, after which eligible clients were enrolled in an ICBT course appropriate to their symptoms and preferences. The current study only included clients enrolled in the English versions of the therapist-guided *PSP Wellbeing Course* or *PSP PTSD Course*.

### Intervention

The *PSP Wellbeing Course* and *PSP PTSD Course* are therapist-guided ICBT courses designed to be completed in 8 weeks. Each is based on ICBT courses, called the *Wellbeing Course* and *PTSD Course*, that were originally developed by the eCentre Clinic at Macquarie University in Australia and have since shown excellent outcomes in Australia [35] and Canada [30]. The *PSP Wellbeing Course* was initially tailored for PSP following a series of interviews, focus groups, and questionnaires exploring their unique needs [3,26] and has since been continuously evaluated and iteratively improved based on client feedback [28]. The *PSP Wellbeing Course* is a transdiagnostic course that is designed to help with symptoms of anxiety, depression, and posttraumatic stress, whereas the *PSP PTSD Course* is a disorder-specific course that focuses on symptoms of posttraumatic stress. Both courses include psychoeducation and coping skills rooted in cognitive behavioral therapy (CBT) and delivered via a combination of text, diagrams, videos, audio files, downloadable practice worksheets and activities, illustrative case stories, frequently asked questions, additional resources addressing issues not addressed in the core course content (eg, problems with anger, alcohol use, and sleep), and optional therapist support via phone or secure email. Each of these courses is described in greater detail in later sections [21,22].

### Measures

During the web-based screening, prospective clients completed a Participant Information Questionnaire that solicited demographic (eg, age, gender, ethnicity, and community size) and occupational data (eg, PSP sector, years of experience, and leadership role). Clients also completed a series of questionnaires assessing symptoms of various mental disorders, which are presented in Table 1. Finally, clients completed a bespoke Treatment Satisfaction Questionnaire at 8 weeks post enrollment. Detailed information about these measures—and several others, not germane to the purposes of this study—can be found in previous publications [21,36] or our open observational trial (NCT04127032) and open cohort preference trial (NCT04335487) registered on ClinicalTrials.gov.

**Table 1:** Sample characteristics (N=10).

Characteristic		Values
Age (years), mean (SD)		45.3 (6.0)
**Gender, n (%)**		
	Women	3 (30)
	Men	7 (70)
**Ethnicity, n (%)**		
	White	8 (80)
	Other	2 (20)
**Community size, n (%)**		
	Less than 100,000 citizens	6 (60)
	More than 100,000 citizens	4 (40)
**Employment status, n (%)**		
	Employed	8 (80)
	Unemployed	2 (20)
**PSP**^a^ **sector, n (%)**		
	Police	5 (50)
	Other	5 (50)
Years in PSP field, mean (SD)		17.2 (6.4)
**Clinically significant symptoms at pretreatment, n (%)**		
	Depression (PHQ-9^b^; cutoff score≥10) [37]	7 (70)
	Anger (DAR-5^c^; cutoff score≥12) [38]	6 (60)
	Anxiety (GAD-7^d^; cutoff score ≥10) [39]	5 (50)
	Posttraumatic stress (PTSD^e^ checklist for DSM-5^f^; PCL-5; cut-off score ≥33) [40]	3 (30)
	2-item PDSS-SR^g^ (cutoff score≥3) [41]	3 (30)
	Social anxiety (Mini-SPIN^h^; cutoff score≥6) [42]	2 (20)
	Alcohol use (AUDIT^i^; cutoff score≥20) [43]	1 (10)
	Drug use (DUDIT^j^; cutoff score≥25) [44]	1 (10)
	Clinically significant symptoms on at least 1 measure	8 (80)
	Clinically significant symptoms on at least 3 measures	5 (50)

^a^PSP: public safety personnel.

^b^PHQ-9: Patient Health Questionnaire-9.

^c^DAR-5: Dimensions of Anger Reactions-5.

^d^GAD-7: Generalized Anxiety Disorder-7 Scale.

^e^PTSD: posttraumatic stress disorder.

^f^*DSM-5*: Diagnostic and Statistical Manual of Mental Disorders (Fifth Edition).

^g^PDSS-SR: Panic Disorder Severity Scale–Self-Report.

^h^Mini-SPIN: Mini-Social Phobia Inventory.

^i^AUDIT: Alcohol Use Disorder Identification Test.

^j^DUDIT: Drug Use Disorder Identification Test.

### Eligibility Criteria

In order to be included in either the *PSP Wellbeing Course* or the *PSP PTSD Course* and, in turn, the study, clients were required to (1) be at least 18 years of age, (2) be a current or former PSP, (3) have internet access, and (4) reside in a Canadian province where PSPNET offers therapist-guided ICBT services (ie, New Brunswick, Nova Scotia, Ontario, Prince Edward Island, Québec, or Saskatchewan). Additional details regarding eligibility criteria are described in previous papers [21,22] and our open observational trial (NCT04127032) and open cohort preference trial (NCT04335487) registered on ClinicalTrials.gov. Further to these criteria, clients were only included in this study if they indicated that they were in a supervisory or leadership role within their public safety organization. Semistructured interviews took place between January 2024 and July 2024.

### Semistructured Interview

Clients were invited via email to participate in a semistructured interview at 6 weeks post enrollment, which allowed 2 weeks to schedule interviews for as early as possible after their completion of the 8-week ICBT programs. Of the 23 clients invited, 10 leaders within public safety completed the interview. One author (JABP) conducted interviews with the first 10 clients interested via phone or video call, with each lasting 20 to 30 minutes. Interview questions were piloted with the first client and then discussed with author (HDH) to ensure clarity and effectiveness before proceeding. Clients provided verbal consent for the interviews and their recordings. First, clients were asked to describe stressors that impact their well-being. Clients were then asked questions assessing their perceptions of and feedback on ICBT: (1) course material, (2) case stories, (3) resources, (4) therapist support, and (5) overall impressions. Given the semistructured nature of the interview, clients were asked follow-up questions where appropriate to gain further insight.

Course material questions assessed which topics and skills were most and least beneficial as a leader in public safety and then solicited suggestions for new topics and skills that would be relevant and beneficial for clients in leadership positions. Case stories questions explored whether leaders accessed at least one of the stories and, if so, whether case stories were perceived as authentic and relatable experiences, beneficial to leaders (ie, informing, comforting, modeling, persuading, and engaging) [45], and whether a leader-specific story is recommended. Resource questions assessed whether leaders accessed at least 1 additional resource and, if so, which resources were perceived as the most and least beneficial for leaders and whether a new leader-specific resource is recommended. Therapist support questions assessed whether leaders accessed the optional therapist support and, if so, preferred modality of therapist support (ie, email or phone), whether therapist support met their expectations, and general feedback. Finally, overall impression questions assessed clients’ satisfaction with their selected therapist-guided ICBT program, perceived benefits and negative impacts of participation, the likelihood of recommending therapist-guided ICBT to other PSPs in both leadership and nonleadership roles, and additional feedback not covered in previous questions.

### Clients

#### Overview

Demographic data were collected via the Participant Information Questionnaire and analyzed using SPSS (version 28; IBM Corp). Results showed that eligible clients predominantly enrolled in the *PSP Wellbeing Course* (n=9, 90%) and self-identified as employed (n=8, 80%), White (n=8, 80%), and men (n=7, 70%) with an average age of 45 years. Half of the clients reported working within policing (n=5, 50%) and half in various other public safety sectors. Clients resided in communities with less than (n=6, 60%) and greater than 100,000 citizens (n=4, 40%) across various Canadian provinces. At pretreatment, 80% of clients endorsed clinically significant symptoms of one or more mental disorders, with 50% endorsing clinically significant symptoms of 3 or more mental disorders. In total, 70% of clients endorsed clinically significant symptoms of depression, 60% endorsed clinically significant symptoms of anger, 50% endorsed clinically significant symptoms of generalized anxiety, 30% endorsed clinically significant symptoms of posttraumatic stress, and 10% endorsed clinically significant problems with alcohol and drug use. Further details surrounding sample characteristics are presented in Table 1.

#### Analyses

Qualitative data (N=10) were sourced from open-ended questions via the semistructured interviews, which were deidentified and transcribed by author SAD. Transcriptions were then analyzed using the NVivo (version 20; Lumivero) analysis software using a reflexive thematic approach. The initial codebook was created by the primary coder, author JABP, to align with questions posed during the semistructured interviews. These themes were then reevaluated to identify, analyze, and report on any subthemes [46]. The authors acknowledge that we hold certain preconceived attitudes and beliefs about ICBT and recognize that, as non-PSP, our understanding of PSP experiences is limited. To help minimize bias, themes were reviewed by author SMS to identify and discuss any conflicts. Themes were then discussed among all other co-authors for diverse perspectives. Upon consensus of coding, each theme was assessed for semantic meaning and given an appropriate name [47]. Client demographics were pulled from the closed-ended survey questions and were imported into IBM SPSS Statistics (version 26) to compute descriptive statistics.

## Results

### Stressors and Reasons for Seeking Treatment

#### Overview

Qualitative analyses identified two reasons why leaders within public safety enrolled in the *PSP Wellbeing Course* (n=9) and *PSP PTSD Course* (n=1): (1) to seek support for their mental health (n=6, 60%) and (2) to review the course for their members (n=4, 40%). Leaders cited two themes of stressors that impact their mental health and well-being: (1) occupational (N=10) and (2) nonoccupational (n=3, 30%). Occupational stressors were further categorized into operational (ie, related to tasks in the workplace; n=7, 70%) or organizational stressors (ie, related to the job setting or context). The operational stressor most reported was workload (n=7, 70%). One client said “The challenge I find as a leader is I work a lot of extra hours” while another stated, “I'm on call 24 hours a day.” Additionally, clients reported operational stress due to PPTE exposures (n=5, 50%). One client explained, “in leadership positions…you get exposed to a lot of things” while another client elaborated, “I’ve been through riots… finding dead bodies.”

Clients further identified a variety of organizational stressors that impact their well-being. Most clients highlighted the continued presence and impacts of mental health stigma (n=7, 70%) within their organizations. One client said, “I’ve been in the service for 29 years and the only place it’s safe to have a meltdown is at home or behind closed doors” while other clients clarified, “Creating a safe space for a leader is really, really difficult because you don’t know who you can trust” and “If you come out and say you have problems, you’re damaged goods in their eyes. So, you keep that to yourself.” Many clients reported a lack of support within their organization (n=6, 60%), stating, “My officers know they can come to me at any time with any issues whether its work or personal. I don’t get that from my leaders” and “There's no [union] representation of being in a management position whereas frontline workers have representation.” Clients indicated concerns related to staffing (n=5, 50%) with one stating, “Most people who would merit that type of a position don’t want the job” and another reporting that “My staff are stretched fairly thin.” Clients reported the impacts of guilt because of their job (n=4, 40%) with one explaining that “You are the ones who are setting up the plans and the ones who are directing people to act and so it's going to be a lot of guilt and trauma.” Another client elaborated “You’re always worried about the safety of the people under you, so you always carry that burden. You want to make sure they’re happy, cared for, looked after, their families are looked after, so you carry that a little bit more.” Clients also highlighted concerns related to interpersonal difficulties (n=4, 40%), “I don’t know how to handle him anymore. I’m beyond being able to deal with him… Dealing with him on a day-to-day basis left me with no energy to deal with stuff at home” and middle management (n=3, 30%), “The stressors come from below as well as above...it comes uphill and it goes downhill and we’re sort of the in between.” Finally, clients identified the impact of resources (n=3, 30%), “I'm burning through cash…I've been addressing some of the staffing issues we have, which has been great, but I've been doing it double time and there's only a finite amount of money.” Nonoccupational stressors were recognized by many, but not all, clients (n=3, 30%) with all identifying familial concerns specific to the death of a loved one (n=1, 33%), interpersonal violence (n=1, 33%), or marital problems (n=1, 33%). More information about these occupational and nonoccupational stressors are discussed in Table 2.

**Table 2:** Occupational and nonoccupational stressors.

Themes			Example quotes	Count, n (%)	
**Occupational stressors**					
	**Operational stressors**				7 (10)
		Workload	“The challenge I find as a leader is I work a lot of extra hours.”	7 (70)	
		PPTEs^a^	“I’ve been through riots…finding dead bodies.”	5 (50)	
	**Organizational stressors**			10 (100)	
		Mental health stigma	“I’ve been in the service for 29 years and the only place it’s safe to have a meltdown is at home or behind closed doors.”	7 (70)	
		Lack of support	“My officers know they can come to me at any time with any issues whether its work or personal. I don’t get that from my leaders.”	6 (60)	
		Staffing challenges	“Most people who would merit that type of a position don’t want the job.”	5 (50)	
		Guilt	“You are the ones who are setting up the plans and the ones who are directing people to act and so it's going to be a lot of guilt and trauma.”	4 (40)	
		Interpersonal difficulties	“I’m beyond being able to deal with him…Dealing with him on a day-to-day basis left me with no energy to deal with stuff at home.”	4 (40)	
		Middle management	“The stressors come from below as well as above...it comes uphill and it goes downhill and we’re sort of the in between”	3 (30)	
		Lack of resources	“I'm burning through cash…I've been addressing some of the staffing issues we have, which has been great, but I've been doing it double time and there's only a finite amount of money.”	3 (30)	
**Nonoccupational stressors**					3 (30)
	Familial		“My wife and I were struggling back and forth, and it has started to affect me, and us, and our kids…and just everything in general.”	3 (30)	

^a^PPTE: potentially psychologically traumatic event.

#### Positive Feedback

Clients generally engaged well with ICBT, as most accessed at least the first 4 of 5 lessons in their selected course (7/10, 70%), completed posttreatment mental health measures (9/10, 90%), and used optional therapist support (7/10, 70%) via phone (3/7, 42%), email (1/7, 14%), or both (3/7, 42%). Clients’ perceptions of ICBT were largely positive. Most clients reported in the treatment satisfaction questionnaire that the course was worth their time (8/10, 80%), that they were satisfied with the course overall (n=9, 90%), and that they would recommend the course to other PSP (N=10). One client stated that “Every lesson has given me something as a first responder leader,” while another recognized that “It is changing my life. I feel less angry and less frustrated.” Of those who had contact with their clinician (7/10, 70%), most clients reported that their therapist support met or exceeded their expectations (6/7, 86%). One client explained, “I think I can be helpful to other leaders if they find themselves in this position.”

Skills most commonly cited as helpful were controlled breathing (6/10, 60%) followed by challenging unhelpful thoughts (4/10, 40%) and graduated exposure (4/10, 40%). Many clients reported positive perceptions of the course delivery (6/10, 60%), highlighting the program’s flexibility (4/6, 67%), structure (2/6, 33%), free-of-cost availability (2/6, 33%), confidentiality (1/6, 17%), and audio features (1/6, 17%). For example, 1 client stated, “You don’t have to go out somewhere to meet with somebody or those kinds of things like for an introvert like me, it’s perfect that it’s like this.”

All clients accessed at least 1 case story (N=10), and most accessed at least 1 additional resource (6/10, 60%). Clients perceived the case stories as authentic representations of PSP (9/10, 90%) that were relatable to their own experiences (9/10, 90%). One client explained, “I’ve seen my staff go through some of the things that they were talking about…So, I could sort of go through the story and empathize with it and possibly be able to use that to help my staff.” Most clients agreed that the case stories were modeled (9/10, 90%) and motivated the use of targeted behavior learned in the course (9/10, 90%). One client stated, “Just reading it…was one thing but then to kind of take those stories and put them into a real-life situation, that absolutely helps.” Clients also described stories as helping to normalize mental health symptoms (8/10, 80%), with 1 client highlighting that “Knowing it’s so common makes it much easier to start facing things.” Moreover, most clients recognized that the case stories increased understanding of and engagement with the course material (7/10, 70%). One client stated, “[The stories] put it into perspective what the material was teaching us so like okay I can see how this applies to a situation or to real life.” Some clients also recognized the case stories as a source of mental health information (4/10, 40%).

### Constructive Feedback

#### Case Stories

Clients provided tangible feedback on ways to optimize therapist-guided ICBT for leaders via the case stories, additional resources, and delivery. Most clients (n=6, 60%) recommended a new story tailored for leaders within public safety. One client stated, “Yeah, it could be beneficial. Especially if…you’re in that middle or senior position.” while another stated “It could be an interesting avenue if you wanted to do a leadership version of this. So, the story is not about you. The story is about somebody on your team.” Two clients were unsure if a leadership story is necessary with one elaborating, “We do have unique issues but there’s also similarities and I think…how they deal with them is going to be the same. So, it’s just that we’re more isolated.” One client recommended removing the stories entirely, stating, “Get rid of them…It makes you feel like – well this person has it worse than you.”

#### Additional Resources

Clients (n=4, 40%) provided topic suggestions for additional resources. Three clients recommended a leadership-specific resource with 2 clients recommending ways to apply the skills learned to a leadership position. Another client requested tools to build confidence, explaining, “For leadership…the huge thing is confidence, self-awareness, and being able to project that confidence and your abilities and skills.” One client also recommended a resilience-based resource that provides tools and skills to maintain mental health. Finally, 1 client suggested an unconscious bias resource, explaining, “Unconscious biases seems to be like a real cancer because everybody has a different one and you don’t know what it is. So, that means everybody’s discriminated against in some way and it makes moving forward in any capacity hard.”

#### Delivery

Additionally, clients (n=7, 70%) provided recommendations for tailoring the course delivery. Three clients highlighted technological challenges in navigating the web-based platform. One client stated, “I don't know if this was myself or the system. The site would load fairly slowly. And it would crash a lot” while another said, “As an older person, technology's not my forte…This isn't just with your course it's with everything.” Two clients suggested incorporating more audiovisuals, with one explaining, “There is sections of the course that are narrated, and they do have someone talking to you and kind of walking you through it, and I liked those parts the best. That’s where I absorbed the most information, I don’t absorb the most if I’m just reading a screen.” Two clients also revealed challenges with the pace of the course, with one explaining “You kind of feel like oh, I should get through this lesson quick because I have another one” and another stating, “It would sure be nice if I didn't have to give the survey on the module I hadn't done yet.”

## Discussion

### Principal Results

#### Overview

ICBT is an effective and convenient mental health treatment among the general population [19,20]. To increase awareness of and access to ICBT, PSPNET was developed with and for PSP across Canada. Previous results show favorable mental health outcomes, treatment engagement, and treatment satisfaction [21,30]. Leaders previously recognized the need and suitability of PSPNET for Canadian PSP and provided advice to optimize implementation generally [32]. This study expands on these findings by exploring ways to optimize the implementation of therapist-guided ICBT specifically among Canadian PSP in leadership roles.

#### Stressors and Reasons for Seeking Treatment

Leaders reported concerns with occupational and nonoccupational stressors impacting their well-being. Occupational stressors included both operational stressors (ie, workload and PPTEs) and organizational stressors (ie, mental health stigma, lack of support, staffing challenges, guilt, interpersonal difficulties, middle management, and lack of resources) [16]. While these findings are broadly consistent with previous research showing the prevalence and diversity of occupational stressors within public safety organizations, it is notable that leaders in the current study described a higher proportion of organizational stressors relative to operational stressors than did a general sample of PSP who were not necessarily leaders in a past study [33]. PSP’s most salient stressors may then shift from operational to organizational in nature as they progress into leadership roles. Some leaders also reported nonoccupational stressors specific to their families. Leaders enrolled in PSPNET most frequently described seeking support for their mental health, with some enrolling instead to review the course for other employees in their organizations. Therefore, despite leadership stereotypes [48], the study shows that Canadian PSP in leadership roles are susceptible to occupational and nonoccupational stressors and may have an interest in seeking therapist-guided ICBT to support their mental health (among other reasons).

#### Positive Feedback

Leaders in this sample showed good treatment engagement and satisfaction, including satisfaction with therapist support, which is consistent with findings of prior research on ICBT tailored for PSP at varying levels of leadership [21,22]. Self-managing mental health symptoms appear to be a priority among leaders just as it is among PSP more broadly [34], although several leaders indicated that they were reviewing the course before recommending it to colleagues. Many leaders in this study cited controlled breathing, challenging unhelpful thoughts, and graduated exposure as helpful skills learned in PSPNET’s courses.

#### Case Stories

Previous research found that many (ie, 69% [34]), but not all, PSP accessed at least 1 case story in ICBT compared to all leaders in this study. Leaders, in general, report increased social isolation [14,15,49] and decreased social support [15] compared to nonleaders. Perhaps leaders are then more likely to engage in ICBT case stories as a result. Nevertheless, most leaders indicated that the case stories represented authentic and relatable experiences [34,50], but still recommended the development of a new story tailored to their needs. Research supports tailoring case stories to current and prospective clientele as it may improve story interest and engagement [51,52]. Therefore, this study highlights the need for and potential benefits of a leadership-based case story in the *PSP Wellbeing Course.* Specifically, content could include: (1) mental health stigma, (2) lack of support, (3) staffing challenges, and (4) guilt, among other topics provided in Table 2.

#### Additional Resources

Leaders similarly recommended the development of a new resource that provides information on the application of the content and skills learned in the course to address their unique stressors. Offering a variety of additional resources provides a flexible way for clients to access psychoeducation and strategies relevant to their needs, without requiring them to seek external support or engage with content irrelevant to their needs [53]. Additional resources can also improve treatment engagement and satisfaction by allowing clients to personalize treatments [53]. This recommendation may then offer promising outcomes given that additional resources are perceived as a valued component of ICBT even though previous research [34] and this study showed that not all clients access them. A leadership-specific resource could support leaders in applying CBT skills to manage the unique issues they identified (eg, how to use thought challenging to address unhelpful thoughts that induce feelings of guilt in leaders and how to use assertive communication skills to manage interpersonal difficulties with staff).

#### Delivery

Leaders continue to recognize the benefits of delivering CBT via a web-based platform. The delivery feature most appreciated by leaders in this study was flexibility, allowing clients to work at their own pace in the privacy of their home or office, with optional therapist support. This finding is consistent with previous research highlighting the need for flexible mental health support for PSP [26,28,32]. For example, McCall et al [26] found that PSPs prefer therapist-guided ICBT that is delivered within a flexible timeframe for course completion and with a choice of communication modes with their assigned therapist. Some leaders also remarked on their appreciation that PSPNET is free and confidential with the accessibility option of audio narratives. To further optimize ICBT delivery, leaders generally recommended improving the platform technology and adding audiovisual content. Technological barriers are a common concern with internet interventions [54] because they play a critical role in treatment engagement and satisfaction [55]. Audiovisual content can also improve treatment engagement and satisfaction by improving accessibility options [56]. Yet, little is known about PSP dropout rates in ICBT and whether they mirror the general population.

### Limitations and Future Directions

This study has several limitations that can help inform future research directions. First, most clients self-identified as White, men, and working in policing. In the largest study of Canadian PSP, we are aware of (N=5813), 92% of the sample identified as White, 67% reported being male, and 52% identified as either municipal or provincial police or Royal Canadian Mounted Police personnel [2], suggesting that our sample is relatively representative of the demographic makeup of Canadian PSP. Nevertheless, our total sample size was very small, and the number of participants who were not White, men, or working in policing was particularly small, limiting the diversity and representativeness of responses and perspectives. Second, sampling bias may be a risk, as clients with positive perceptions of PSPNET may have been more likely to complete questionnaires and agree to participate in interviews. As such, clients with negative perceptions of PSPNET may not have been included in this study. Additionally, this study did not consider the role of mental health history (symptoms or services) or type of leadership role (eg middle or upper management) on leaders’ perceptions of ICBT. Future research could include a larger sample size to improve sample diversity with a particular focus on sector, gender, ethnicity, mental health history, and types of leadership roles. Quantitative analyses could then explore the role that demographics play in leaders’ preconceived beliefs about ICBT. A follow-up thematic reflexive analysis is also recommended to investigate the impact of a leader-specific case story and additional resources on ICBT engagement, mental health outcomes, and satisfaction.

### Conclusions

This study provides insight into the suitability and optimization of therapist-guided ICBT for Canadian leaders within public safety. Most leaders reported enrolling in PSPNET for support with occupational and nonoccupational stressors impacting their well-being and symptoms of depression, anger, anxiety, posttraumatic stress, and other concerns. Some leaders reported enrolling in PSPNET to review the course for their staff. Nevertheless, most leaders held positive perceptions of therapist-guided ICBT and provided tangible feedback to further optimize its implementation, focusing on the case stories, additional resources, and delivery. Overall, these findings are promising given the important role that leaders play in the promotion, implementation, and sustainability of programs and services [9,57].

## Abbreviations

CBT: cognitive behavioral therapy
ICBT: internet-delivered cognitive behavioral therapy
PPTE: potentially psychologically traumatic event
PSP: public safety personnel
PTSD: posttraumatic stress disorder

## Data Availability

The data described in this study are not publicly available due to a commitment to the participants to securely store their data and refrain from sharing them with anyone outside of the research team.
